# Intraparenchymal Renal Artery Pseudoaneurysm and Arteriovenous Fistula on a Solitary Kidney Occurring 38 Years after Blunt Trauma

**DOI:** 10.1155/2017/3017501

**Published:** 2017-03-13

**Authors:** Daniel Benamran, Benedicte de Clippele, Frank Hammer, Bertrand Tombal

**Affiliations:** ^1^Department of Urology, Geneva University Hospitals, 4 rue Gabrielle Perret-Gentil, 1205 Geneva, Switzerland; ^2^Department of Urology, University Clinics Saint Luc, Catholic University of Louvain, 10 Avenue Hippocrate, 1200 Brussels, Belgium; ^3^Department of Radiology, University Clinics Saint Luc, Catholic University of Louvain, 10 Avenue Hippocrate, 1200 Brussels, Belgium

## Abstract

Pseudoaneurysm and arteriovenous fistulae of the renal artery are rare complications of kidney trauma. They commonly result from open traumas and occur within days after the injury. Common symptoms include acute haematuria, pain, or hypertension. We report the case of a fifty-three-year-old man presenting with symptomatic complex chronic high flow kidney arteriovenous fistula with interposition of a pseudoaneurysmal pouch and arterial aneurysmal dilatation in a solitary left kidney 38 years after a blunt trauma. Those conditions were successfully treated by endovascular embolization followed by regular radiologic, biological, and clinical follow-up. To the best of our knowledge, few similar cases were reported more than 20 years after trauma. However, no case combining an arteriovenous fistula and a pseudoaneurysm revealing as late as 38 years after trauma was found. In addition, management of those conditions on a solitary kidney and outcomes has not been described. We believe that our case depicts the clinical presentation and management of this rare entity that should not be unrecognized due to its potential lethal implications.

## 1. Introduction

Renal artery aneurysmal anomalies are uncommon. The estimated incidence in the population ranges from 0,01% to 1% [1]. It accounts for 22% of all visceral aneurysms [2], with only 17% being intrarenal. While most of them are asymptomatic their clinical manifestations can include hypertension, haematuria, and pain. The onset of these may be brutal while occasional rupture may lead to more deleterious consequence [3]. Arteriovenous fistulae are also rare most of them being consequences of an iatrogenic injury such as a biopsy, a nephrostomy, a nephrolithotomy, or a partial nephrectomy. Blunt abdominal trauma is a rarer cause of fistula [4], with symptoms occurring days or weeks after the traumatism [5]. The standard treatment of renal arteriovenous fistulae and arterial aneurysmal anomalies in a stable patient is selective arterial embolization [6]. To the best of our knowledge, only a few cases of posttraumatic arteriovenous fistula occurring more than 20 years after the trauma have been described [7–9], all of them occurring on patients having both their kidneys. We report the case of a man diagnosed 38 years after blunt abdominal trauma with a renal artery pseudoaneurysm and an arteriovenous fistula occurring on a solitary kidney.

## 2. Case Report

A fifty-three-year-old man presented with acute left flank pain while eating dinner. The only noticeable medical history was that he underwent, 38 years ago, a right nephrectomy for a blunt renal trauma when falling from a ladder on the right flank while cleaning gutters outside his house. He also reported hypertension well-controlled with 5 mg amlodipine and obesity (body mass index 33). Familial history was negative. Of particular interest, he never reported haematuria or any other urinary symptom. Clinical examination at admission in the emergency department was strictly normal.

**Figure 1:**
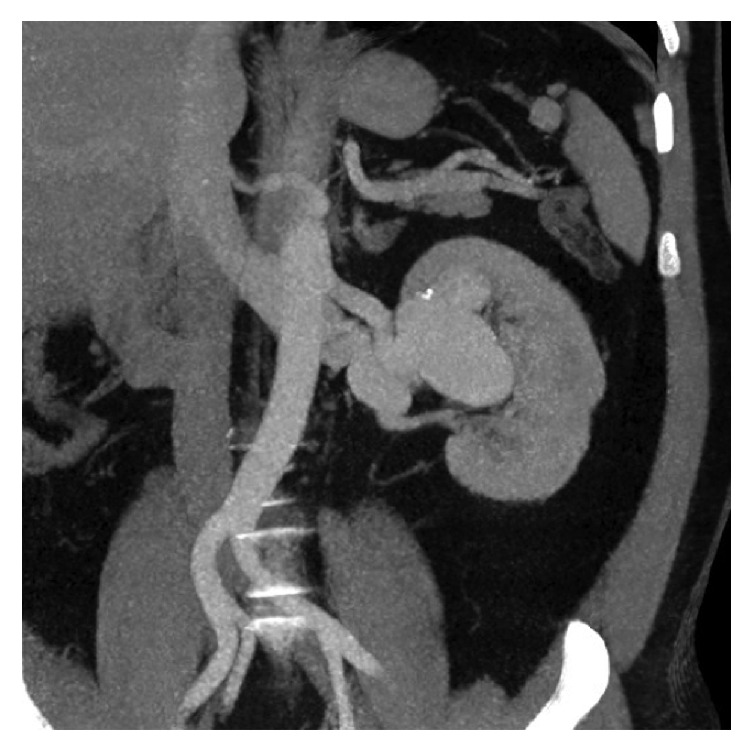
Admission CT-scan showing complex vascular intraparenchymal renal anomaly.

**Figure 2:**
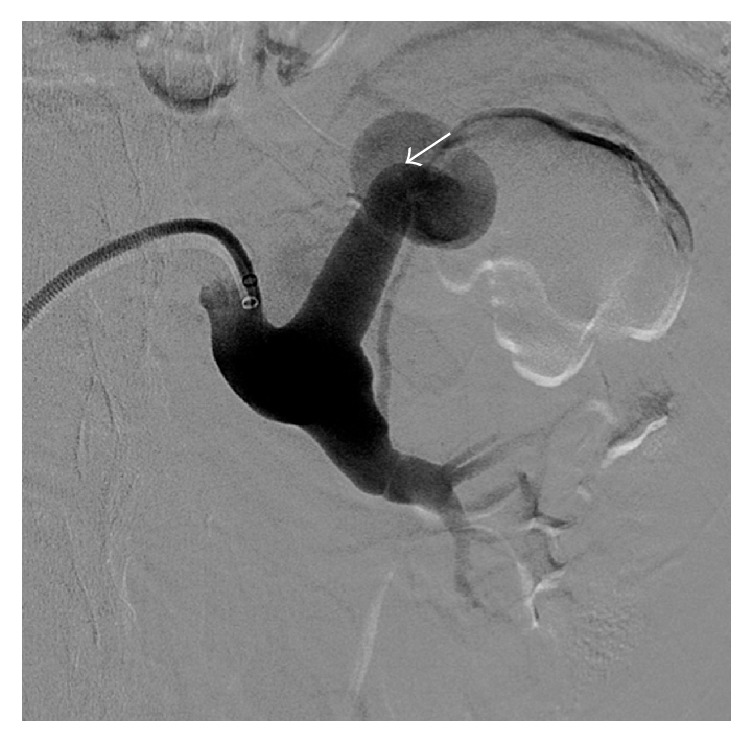
Digital subtracted angiography at early arterial phase showing an hypertrophic aneurysmal left renal artery. White arrow points to a aneurysmal dilatation at the level of the arteriovenous fistula corresponding to the interposition of a chronic pseudoaneurysm between the upper segmental artery and the draining vein.

**Figure 3:**
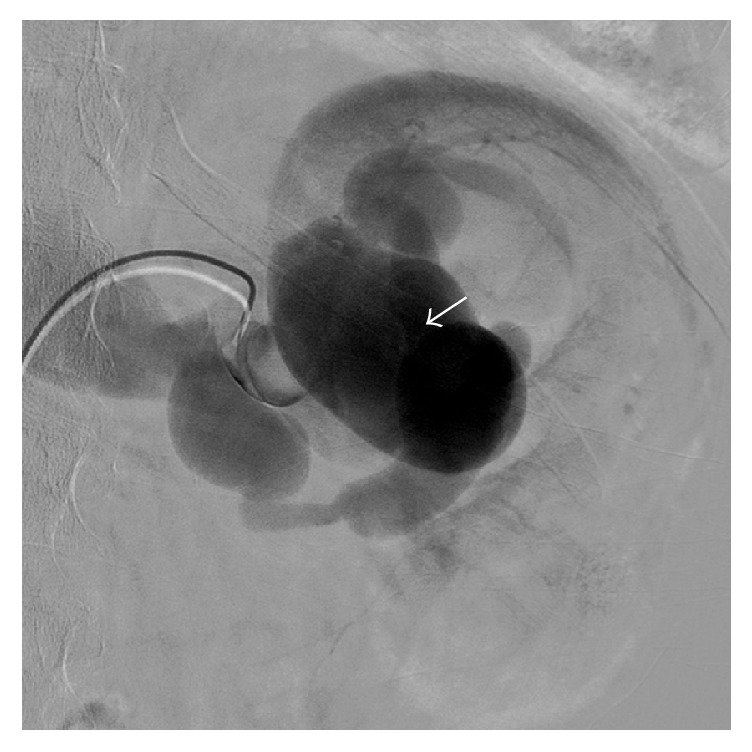
Digital subtracted angiography at the venous phase showing multiple dilatations of the draining renal veins, especially one huge venous dilatation (white arrow) with interposition of a normal segment before drainage into the left renal vein.

**Figure 4:**
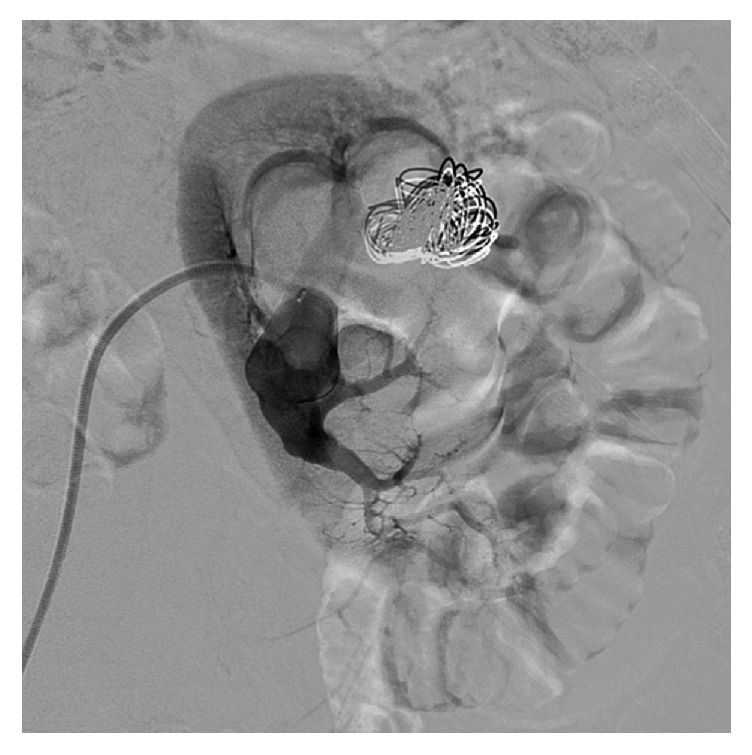
Control angiogram after embolization.

The laboratory examination was normal, without anaemia (haemoglobin 14.2 g/dl, normal range 12–16 g/dl) or renal insufficiency (serum creatinine 0.99 mg/dl, normal range 0.6–1.3 g/dl). The urinary sediment was normal. Since the pain persisted, ultrasound of the abdomen was ordered which revealed a complex vascular anomaly. Diagnostic work-up was completed with computed tomography of the abdomen (Figure 1) which confirmed a complex vascular anomaly with a huge left renal artery. Patient was immediately referred for diagnostic and therapeutic angiography. Digital subtracted angiography demonstrated a high flow fistula, which explained the aneurysmal dilatation of the left renal artery including the superior interlobular artery and aneurysmal dilatations of the renal draining vein. At the arterial phase, the enlarged interlobular artery directly drains through the arteriovenous fistula into a first cyst-like cavity (20 mm) corresponding to a chronic arterial pseudoaneurysm (Figure 2). This cavity has been used to trap the coils during embolization, preventing their venous or pulmonary migration. At the venous phase a huge venous dilation is seen (54 × 37 mm), subsequently draining into a smaller venous dilatation and from there into the main renal vein (Figure 3). Six Azur-35 coils (Terumo) were used (15 × 20, 12 × 20, 12 × 15, 10 × 20, 10 × 15, and 8 × 15) and one Interlock-35 (Boston Scientifics) 12 × 40. Immediate and complete occlusion was shown by the control angiogram (Figure 4).

Pain resolved immediately after the procedure and patient was discharged the following day.

Two-week follow-up abdominal ultrasound showed a segmental upper and middle pole hydronephrosis. MAG3/Lasix renal scintigraphy confirmed the segmental obstruction with significant urodynamic obstacle managed conservatively. Follow-up MRI at 3 months showed successful embolization with no recurrence of the lesions but persistent hydronephrosis due to external compression by the aneurysmal thrombotic sac. Since the patient remained asymptomatic and renal function unaltered, a simple follow-up was decided after discussion. At this point indeed, the risk of a complex vascular surgical reconstruction on this solitary kidney outweighs the excepted benefits, especially since the risk of aneurysmal rupture was no longer significant.

At 6 months follow-up, patient was still asymptomatic, kidney function was unaltered, and renal ultrasound did not show any loss of renal parenchyma.

## 3. Discussion

Renal artery aneurysms are uncommon and usually extrarenal. Most of them are attributed to fibroplasia and medial degeneration because the most common histopathological finding is fragmentation of the internal elastic lamina [6]. They are of mixed origin and may be congenital, posttraumatic and iatrogenic, or associated with systemic diseases such as neoplasms or polyarteritis nodosa. They have most commonly been reported in relation to iatrogenic causes such as open or percutaneous surgery or biopsy [10] and are exceedingly rare after a nonpenetrating trauma. Most of them are asymptomatic. With the rare exceptions of dramatic bleeding, their most frequent manifestations are hypertension or flank pain. The natural history of these aneurysms slightly differs from other visceral aneurysms with a lower but not inexistent risk of rupture [6]. This risk increases if the diameter of the aneurysm exceeds 1 cm. The risk of rupture of a pseudoaneurysm is even higher that of an aneurysm of comparable size due to the poor support of the pseudo-aneurysmal wall, since pseudoaneurysms are formed between the two outer layers of the artery while aneurysms involve all three layer of the artery. Active treatment is often indicated in case of clinical symptoms and renal dysfunction or if the aneurysm grows too big [2].

Arteriovenous fistulae are also a rare complication of renal injury, which commonly presents within weeks after the trauma. Blood flows under pressure from the renal artery and pushes the surrounding renal parenchyma open, creating a cavity varying in size and shape [10]. Fistulae are generally caused by a penetrating trauma but can also have a congenital or idiopathic origin. Dilatation of the drainage system is not uncommon after an arteriovenous fistula. Diagnosis is based on angiographic evaluation when a renal or renovascular disease is suspected. Depending on the location of the fistula a murmur can sometimes be heard at physical examination, which is a manifestation of turbulent blood flow in the fistula.

Historically, surgery (e.g., partial nephrectomy or vascular reconstruction) was the only treatment option for these vascular anomalies. The main drawback of these procedures was the important parenchymal loss and nephrectomy being always a possible outcome. Although ex vivo surgical repair is feasible, endovascular embolization has emerged as be the preferred way to preserve renal parenchyma while assuring a significant success rate of 90% after first treatment, especially for intraparenchymal aneurysms [6]. Major contraindications to endovascular procedures include untreated coagulopathy or the haemodynamically unstable patient's condition. Radiological follow-up is imperative and rare complications include coil's migration, accidental embolization of the wrong arterial branch, and dissection of the renal artery.

In a review by Lindekleiv et al. [9], 75% of the patients with renal pseudoaneurysm consecutive to blunt trauma presented with haematuria and 35% with pain. Only a few cases after blunt trauma presented as late as after 15 years. Wang et al. [7] and Lesaux et al. [8] described two other cases presenting 20 years after trauma but were caused by gunshot injuries. We believe that patients presenting with any of the compatible symptoms should always been asked for thorough medical history, especially since these potentially lethal vascular anomalies can easily be missed or misdiagnosed on primary evaluation and can be effectively treated.

In addition, our case highlights the difficulty of managing these conditions on a solitary kidney with urodynamically significant segmental hydronephrosis after primary treatment. Expectant management could lead to partial parenchymal atrophy, but active reconstruction could possibly lead to the loss of the whole kidney. No evidence can be found in the literature to support any of the options and decisions should be made according to patient's preference after consilium by an experienced multidisciplinary team of radiologists, urologists, and vascular surgeons.

## 4. Conclusion

Posttraumatic arteriovenous fistulae and renal segmental pseudoaneurysm are rare but potentially severe conditions that can occur exceptionally decades after the initial injury. When suspected, arteriography should be done to confirm diagnosis and attempt endovascular treatment. Management and follow-up should be planned on a case-by-case basis.
